# Feasibility of Precision Medicine in Hypertension Management—Scope and Technological Aspects

**DOI:** 10.3390/jpm12111861

**Published:** 2022-11-07

**Authors:** Meir Nitzan, Itamar Nitzan

**Affiliations:** 1 Department of Physics/Electro-Optics Engineering, Lev Academic Center, 21, Havaad Haleumi Street, Jerusalem 9116000, Israel; 2 Neonatal Department, Shaare Zedek Medical Center, Shmu’el Bait St. 12, Jerusalem 9103102, Israel; 3 Faculty of Medicine, Department of Pediatrics, The Hebrew University of Jerusalem, Ein Kerem, Jerusalem 9112102, Israel

**Keywords:** blood pressure, hypertension, precision medicine, accurate measurement, blood pressure variability, arterial stiffness, photoplethysmography

## Abstract

Personalized management of diseases by considering relevant patient features enables optimal treatment, instead of management according to an average patient. Precision management of hypertension is important, because both susceptibility to complications and response to treatment vary between individuals. While the use of genomic and proteomic personal features for widespread precision hypertension management is not practical, other features, such as age, ethnicity, and cardiovascular diseases, have been utilized in guidelines for hypertension management. In precision medicine, more blood-pressure-related clinical and physiological characteristics in the patient’s profile can be utilized for the determination of the threshold of hypertension and optimal treatment. Several non-invasive and simple-to-use techniques for the measurement of hypertension-related physiological features are suggested for use in precision management of hypertension. In order to provide precise management of hypertension, accurate measurement of blood pressure is required, but the available non-invasive blood pressure measurement techniques, auscultatory sphygmomanometry and oscillometry, have inherent significant inaccuracy—either functional or technological—limiting the precision of personalized management of hypertension. A novel photoplethysmography-based technique for the measurement of systolic blood pressure that was recently found to be more accurate than the two available techniques can be utilized for more precise and personalized hypertension management.

## 1. Introduction

The mainstay of clinical management of a disease is to provide the patient with the best treatment associated with minimal adverse events. Owing to the complexity of the human body and the diversity of dysfunctions, the approach of “one dose for all” or “one-size-fits-all” that determines clinical decisions according to an *average* patient cannot provide the most efficient clinical care to the individual patient. Personalized medicine or precision medicine (PM) stratifies patient groups according to relevant features of their profile, thereby facilitating more precise management to the individual patient. The personal features of the patient may include genomic, proteomic, metabolomic (“-omics”), and clinical and physiological information, which are relevant to the medical problem [1,2,3]. The recent development of novel diagnostic tools and genomic and proteomic techniques enables the achievement of comprehensive overview of the patient’s profile, facilitating enhancement of the precision of the management. In particular, the advancement in the technology of genomic sequencing and the resultant decrease in its costs – a genome can be sequenced for approximately 1000 USD—facilitates personal genomic tests for directing the treatment [4]. Another technological advancement that is imperative for the progression of PM is the development of efficient machine learning tools capable of analyzing the huge amount of patients’ features data and their association with the different options of management [1,2,5].

Most of the published PM studies relate to oncology, utilizing recent technological and analytical advances in molecular genetic characterization of patient tumors. Those studies contributed to better understanding of the patient-specific mechanisms of the malignancy, more accurate diagnosis and prognosis, and the potential for more precise management, in particular the development of more efficient drugs [1,5,6,7,8,9,10]. Some of those studies led to clinically validated FDA-cleared tests or laboratory developed tests. PM studies have also been performed in non-cancer diseases, leading to the application of omics analyses to prevent and treat cardiovascular diseases, diabetes, tissue rejection of heart transplant, rheumatoid arthritis, Alzheimer, hypertension, and ALS [1,8,9,10,11,12,13,14,15,16,17,18]. The current article examines the feasibility of implementing PM in hypertension management.

## 2. Scope of Personalized Hypertension Management

Implementation of genomic analysis in clinical personalized management of hypertension has not yet been established. Large-scale genomics studies, each examining over 750,000 people, identified hundreds of loci associated with blood pressure (BP) traits [19,20]. Studies on pharmacogenomic (drug–gene) interactions associated with the efficacy of antihypertensive medications have also been performed, but the outcomes were not definite; while some genes were found to be associated with adverse cardiovascular outcomes and various antihypertensive drugs in several studies, in other studies, those associations were not found [21]. In addition, there were no statistically significant interactions in a large study that investigated the interaction between four antihypertensive drugs and single nucleotide polymorphisms and its associations with adverse cardiovascular outcomes [21,22].

In present practice, utilizing the available genomic techniques for personalized management of hypertension might be feasible for selected groups of patients, but it seems to be impractical for the wide population of hypertensive patients. The high prevalence of hypertension (31% or 46% of adults above 45 years in the U.S. population, depending on the recommended threshold for hypertension [23]) and the low hazard rate of hypertension for adverse events, as compared with that of cancer, might not justify the present costs of using genomic sequencing for hypertension management—approximately 1000 USD for each examination. At present, large-scale clinical genomic-based management of hypertension cannot be performed, because of its need for specific infrastructure and resources for genomic tests that are not available everywhere, particularly in low- and middle-income countries [10]. Hence, the PM approach in hypertension management should be based on phenotype biomarkers related to the origin and outcomes of hypertension and to neural and humoral BP regulation.

In fact, some of the recommendations in the guidelines for the definition of hypertension threshold, the target for treatment, and the treatment itself are based on information of the individual patient, and the recommendations differ according to age, ethnicity, and comorbidities. In the 2017 ACC/AHA Guidelines [24], the threshold of systolic and diastolic blood pressure (SBP and DBP, respectively) for treatment by BP-lowering medications is 140/90 mmHg for adults with no history of cardiovascular disease (CVD) *and* with an estimated 10-year risk lower than 10% of atherosclerotic cardiovascular disease (ASCVD), and 130/80 mmHg for patients with clinical CVD *or* adults with an estimated 10-year ASCVD risk of 10% or higher. The selection of drug class also depends on the cardiovascular and other comorbidities of the patient, such as kidney disease and diabetes (see Table 18 in the guidelines [24]). Personalized precision medicine will utilize many more BP-related clinical and physiological characteristics in the patient’s profile for the determination of the BP threshold, target for treatment, and suitable medication. In order to account for all relevant information, machine learning techniques are required [2,25].

Gender is a personal feature of particular importance for BP management, which is often missed in guidelines for hypertension management. As the trajectories of BP change with age differ between men and women, and hypertensive women are more prone to develop hypertension-related CVD, gender should be considered in personalized hypertension management [26,27,28].

The characteristics in the patient’s profile that are relevant to the BP value can be obtained from the medical records of the patient and by biochemical and physiological laboratory testing. Potential biochemical biomarkers related to atherosclerosis can be found in the literature [29,30] and will not be dealt with in the current perspective. At present, the SBP and DBP values are the only cardiovascular parameters utilized for the diagnosis and treatment of hypertension, and adding other relevant cardiovascular features to the diagnosis of hypertension is expected to significantly benefit its management [31,32]. It has been shown that the BP-lowering treatment strategy, based on cardiovascular risk assessment, could prevent more events than the traditional approach that only relies on BP levels [32]. Specific physiological and biochemical features that are greatly related to hypertension are peripheral arteriolar resistance [33,34], arterial stiffness that can be increased by arteriosclerosis, elevated sympathetic activity, renin-angiotensin system, and increased body fluid volume that is related to excess sodium intake [35,36,37]. Blood pressure variability and instability that are related to sympathetic dysfunction also have important role in the progression of CVD and hypertensive emergency [31,38,39]. In general, blood pressure variations due to seasonal, diurnal, and other factors are also related to CVD and should be determined [39,40], as well as the difference between blood pressure measurements at the clinic and at home [41,42]. Dinstag et al. [43] analyzed the data of the SPRINT Study [44] and ACCORD Study [45] and showed that adding the longitudinal BP measurements obtained during the follow-up of the two studies to those collected upon recruitment contributed to more precise risk estimation.

Messerli [35] proposed in 1981 personalized treatment of hypertension management. He recognized that high blood pressure is not a homogeneous disease but is associated with varying mechanisms according to the stage of the hypertension, and suggested that the choice of antihypertensive drugs should be based on clinical findings related to pathophysiologic changes observed during evaluation of hemodynamic, fluid volume, and endocrine data. While implementing the recommendation was not feasible at that time because of paucity of diagnostic tools and a lack of computerized techniques for large-scale data analysis, it seems that the current advancement in both technological areas can be utilized for more precise management of hypertension.

Both hypertension and hypotension are associated with the risk of severe morbidities. Hypertension is related to CVD risk that might be decreased by intensive treatment, while hypotension (which can be caused by the intensive antihypertensive treatment) can result in reduced perfusion to the brain and kidneys, which might lead to syncope, kidney dysfunction, poor physical and cognitive functions, dementia, and mortality in elders [23,43,44,46,47]. The expected risk of CVD and other adverse events should also be considered as data that might assist clinicians to manage hypertension with greater precision, say, to provide the patient with better treatment associated with minimal adverse effects.

## 3. Technological Aspects of Personalized Hypertension Management

### 3.1. Selection and Evaluation of Biomarkers

In personalized precision management of hypertension, one makes clinical decisions according to individual relevant patient features rather than according to an *average* patient, with expectations to provide better management. Personal features, such as age, ethnicity, and cardiovascular diseases, have already been addressed in guidelines for hypertension management and, in precision medicine, additional clinical and physiological characteristics in the patient’s profile, which are related to blood pressure, can be utilized for optimal management. It should be noted that laboratory-obtained clinical biomarkers were also applied in recent guidelines (e.g., [24], Table 17) to facilitate CVD risk factor profiling and establish a baseline for medication use.

As was mentioned above, Messerli [35] proposed personalized treatment of hypertension in 1981, realizing that hypertension is associated with several mechanisms. He suggested that the choice of antihypertensive drugs will be based on measurements of hemodynamic parameters, fluid volume, and endocrine data, but at that time, there were not sufficient diagnostic tools and computerized techniques for the measurements of the physiologic features and for the analysis of the diagnostic data. The recent advancement in the development of tools for the assessment of cardiovascular, neurological, and humeral characteristics related to the development of hypertension, as well as the analysis of relevant data, enable more precise management of hypertension.

The diagnostic tools for the measurement of the characteristics that can be used in personalized hypertension management are dictated by the high prevalence of hypertension and the relatively low hazard rate of hypertension for adverse events (as compared with that of cancer and diabetes). The tools should be non-invasive and simple to use by medical staff in hospitals and community clinics. In the following, we refer to several hypertension-related parameters that can be estimated by non-invasive and simple techniques.

**Figure 1:**
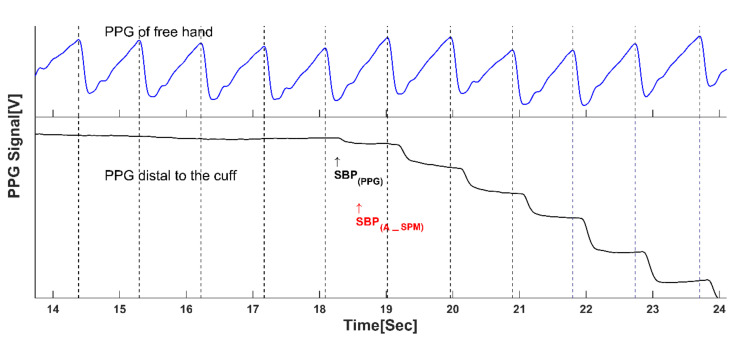
Curves of the PPG in fingers in the two hands at a time interval during cuff deflation, when the cuff-pressure was in the neighbourhood of the SBP value. The blue curve is the PPG signal measured in the cuff-free hand. The black curve is the PPG signal measured in the hand distal to the cuff. When the cuff air-pressure decreases below SBP, the arteries open and the PPG pulses reappear. At the same time, the baseline of the PPG curve starts to decrease because the cuff-pressure is above the venous pressure, and blood flowing through the arteries is accumulated in the veins, thereby reducing the light transmission through the tissue. The start of each PPG pulse in the free hand (at end-diastole/start of systole) is marked by a vertical line, serving as a time-reference for validating that the pulse in the black curve is a PPG pulse. The arrows marked by SBP_(PPG)_ and SBP_(KA)_ designate the time at which the first PPG pulse and the first Korotkoff appear, respectively.

Arterial stiffness and peripheral arteriolar resistance, the main physiological features that increase blood pressure, can be increased by arteriosclerosis, elevated sympathetic activity that increases the tonus of the arterioles, renin-angiotensin system, and increased body fluid volume. Arterial stiffness of the aorta is the main cause of systolic hypertension and it can be estimated by measurements of carotid-femoral pulse wave velocity (PWV) [48,49,50] or analysis of the blood pressure pulses, employing applanation tonometry [51,52]. Peripheral PWV, such as brachial-ankle, femoral-ankle, and carotid-radial, can be used for the assessment of arterial stiffness [50,53]. The PWV measurements can be performed by a tonometer, which measures blood pressure waves; Doppler ultrasound; or peripheral photoplethysmographic (PPG) pulse [54]. PPG is the measurement of the oscillations in the transmitted light through tissue (Figure 1), which are caused by the cardiac-induced oscillations in arterial blood volume (PPG in two wavelengths is the basis for pulse oximetry for the measurement of arterial oxygen saturation). Charlton et al. [55] presented several PPG-derived parameters related to arterial stiffness and other features of vascular age. PPG is very simple to use, in contrast to the non-invasive measurements of arterial blood pressure waves and Doppler ultrasound velocity, in which operator skill is required to operate the device and the level of expertise might influence the readings.

Total peripheral resistance is the ratio between arterial blood pressure and cardiac output and the peripheral resistance of the tissue in an organ is the ratio between arterial blood pressure and the blood flow to the organ [56]. At present, a non-invasive and convenient technique for the measurement of cardiac output is not available, but blood flow to a specific tissue can be measured by laser Doppler (in the microcirculation) [57] or by Doppler ultrasound (in a large artery) [56]. Both techniques are not accurate, but their use in personalized hypertension management can provide added value. Another parameter related to the blood flow to the tissue is the ratio between the pulsatile component of the PPG pulses and the mean value of the pulse, which is relatively constant and changes slowly (generally referred to as AC and DC, respectively, see Figure 1) [58,59]. The parameter AC/DC is suggested by Massimo (a company that produces pulse oximeters, Irvine, CA, USA) as the perfusion index, a proxy to blood perfusion [60,61].

Sympathetic nervous system (SNS) activity is a physiological parameter that is also relevant to personalized hypertension management, because SNS is involved in the regulation of heart activity, arterial blood pressure, and blood flow. SNS activity can be estimated by several parameters of the spontaneous variability in heart rate [62,63,64] and by changes in heart rate and blood pressure after changing position and after performing various maneuvers [64,65,66]. Heart rate and heart rate variability can be measured by either ECG or PPG. The very low fluctuations in PPG amplitude and baseline are correlated with the very low fluctuations in SBP and DBP [67] and can thus serve as a blood-pressure-related parameter.

### 3.2. Accurate Measurement of Blood Pressure

The measured SBP and DBP values are the mainstay of the current guidelines of hypertension management, and it is expected that they would be the same in the personalized management of hypertension. As the aim of the latter is to provide hypertension management with greater precision, the issue of the inaccuracy of the BP measurement by the available techniques should be considered. The two available techniques for BP measurement are the manual-auscultatory sphygmomanometry (SPM), which must be used by a skilled examiner at an office or clinic, and the automatic oscillometry, which can be used at home and is also recommended to be used at an office and clinic. The automatic oscillometry can be used in single measurements as well as in 24 h ambulatory BP monitoring.

Auscultatory SPM is considered more accurate than oscillometry, when both are compared to the gold standard, invasive intra-arterial BP measurement, and is regarded as the validation reference of automatic BP meters, mostly oscillometric. However, the auscultatory SPM has to be performed manually by a trained examiner, leading to falsely high BP readings in some examinations owing to white-coat hypertension. In addition, the office BP measurements performed at a few single time points do not fairly represent the average BP value, which has significant variability with time [31,38,39]. It is accepted that office BP measurements have limited sensitivity and specificity in the diagnosis of hypertension [24,68].

Oscillometry, the automatic technique for BP measurement, is based on the measurement of the air-pressure oscillations in the pressure-cuff (which are induced by the heart-beat) during its deflation and the analysis of the increase in the oscillations to a maximal value and subsequent decrease. The determination of the SBP and DBP from the oscillometric pattern is performed through various empirically derived algorithms [69,70], and is thus prone to significant errors [71,72,73,74]. The low accuracy of oscillometric devices can be deduced from the standards imposed by the organizations AAMI/ESH/ISO [75]. An automated blood pressure meter can meet these standards even if its readings deviate from those of the validation reference (the auscultatory technique) by 16 mmHg or more in 5% of the examinations and by 10 mmHg or more in 18% of the examinations [76,77]. It should be noted that the readings by the auscultatory SPM also have a measurement error and they underestimate SBP as measured by the gold-standard invasive BP measurement [78,79,80,81]. Despite their low accuracy, oscillometric BP measurements are recommended by most guidelines, e.g., [24,82,83], because the error in the office BP measurement by the manual-auscultatory technique is greater. The accuracy of the oscillometric BP measurements can be increased by multiple measurements and in particular by 24 h ambulatory BP monitoring (ABPM). Both ABPM and multiple BP measurements at home (HBPM) are considered as more accurate than single office measurement, and either of them is recommended for confirmation and management of hypertension [24]. Nevertheless, routine wide use of ABPM for the general population is less practical because of its inconvenience and cost.

Besides the available techniques, auscultatory SPM and oscillometry, there is also another technique for SBP measurement, based on photoplethysmography (PPG), that is still under development, but has already been found to be significantly more accurate than oscillometry and even more accurate than the auscultatory SPM [77,84]. The PPG-based technique thus has the potential to have the essential accuracy required for precision management in hypertension.

The PPG signal consists of oscillations in light transmission through a tissue, owing to greater light absorption during systole, when the blood pressure and blood volume in the tissue arteries increase (Figure in the Appendix, upper curve. The pulses in the PPG signal are the basis for pulse oximetry, which measures arterial oxygen saturation). The PPG-based technique for the measurement of SBP consists of two PPG probes on fingers of the two hands and a pressure-cuff wrapped around the arm. When the air-pressure in the cuff increases above SBP, the brachial artery under the cuff collapses and the PPG pulses in the finger distal to the cuff disappear. During cuff deflation, the PPG pulses reappear when the cuff pressure decreases below the SBP value (Figure 1). Hence, the cuff pressure in which the PPG pulses reappear is the SBP value. The pulses in the PPG signal in the other hand are used to validate, by timing, that the pulses in the light transmission curve in the finger distal to the cuff are PPG pulses. The PPG-based technique can provide the accurate SBP measurement required for effective precision hypertension management.

## 4. Discussion

The application of PM for the management of hypertension should be based on the selection and acquisition of clinical, physiological, and genetic features that are relevant to hypertension and making use of them to provide more precise management to the individual patient. At present, there is not yet an agreement regarding the genes that are relevant to hypertension [21,22], as described in the Introduction, and even when some information is known, the current high cost and low availability of genomic sequencing hinder their application in widespread hypertension management [10], considering the high prevalence of hypertension in the adult population and the need for specific infrastructure for the genomic tests. Hence, personalized hypertension management should be based on phenotype biomarkers, which affect or are affected by SBP and DBP, and are also accessible from the aspects of cost and need for specific skill in order to allow widespread use of the management.

The management of hypertension includes initial diagnosis that should be established in the clinic and follow-up that can be carried out either at home or at clinic. At present, the follow-up at home includes only automatic SBP and DBP measurements, which can be performed several times a day, several days a week, enabling the reduction in the BP variability effect (besides the advantage of eliminating the white coat and masking effects). The clinical biomarkers obtained in laboratory and required for personalized hypertension management cannot be obtained at home, but their occasional clinic measurement is sufficient. In order to use the personalized hypertension management approach at home in the follow-up period, it would be necessary to monitor some of the relevant physiological characteristics by means of automatic devices that are simple to use and not expensive. In recent years, the PPG has been used for the development of several techniques that enable evaluation of several cardiovascular features as well as neurological features that affect the blood pressure [55,58,59,67]. The PPG components are not expensive and the PPG technology can be easily implemented in simple medical devices that can provide individual information relevant to hypertension treatment in clinics and at home, using self-measurement or tele-medicine.

The measurement of blood pressure is the core of hypertension detection and its treatment and the low accuracy of the available BP measurement techniques limit the precision in personalized hypertension management and might hinder its progress. The use of ABPM might be an acceptable solution, but the technique is inconvenient and costly and is not suitable to widespread management. The PPG-based technique for the measurement of SBP, which was found to be significantly more accurate than the available oscillometric technique [77,84], is automatic and simple to use and can provide the required accurate SBP readings for precision hypertension management. The technique requires two PPG probes in both hands, which can also be used for the acquisition of some physiological features for personalized hypertension management.

The medical problem of hypotension is as important as hypertension and both of them lead to cardiovascular adverse events. While hypertensive patients have greater risk for cardiac infarct and stroke, hypotension results in hypo-perfusion to the vital organs, in particular the brain, which might lead to falls and syncope [44,85], renal disease [44,86], and cognitive impairment and dementia [47,87,88,89]. Both personalized/precision hypertension management and more accurate blood pressure measurement can reduce the risk of hypotension during pharmacological treatment of hypertension.

Implementation of a personalized hypertension management approach poses a problem regarding the suitability of the present guidelines to the new situation in which the tool of personalized hypertension management is implemented. The present guidelines are based on selecting several groups according to a few personal features and providing them with different guidelines; personalized hypertension management will need new guidelines, which will be based on the utilization of a greater number of personal clinical and physiological features, probably based on machine learning. Furthermore, the present guidelines for hypertension management only aim at lowering the blood pressure, while hypertension management based on additional information about relevant personal clinical and physiological features should also aim at changing modifiable features, such as arterial compliance or fluid volume.

## 5. Conclusions

At present, the personalized hypertension management approach should be based on phenotype biomarkers related to hypertension and its regulation. Owing to the high prevalence of hypertension, the techniques for measuring those biomarkers should be non-invasive and simple-to-use by medical staff in hospitals and community clinics, and several relevant techniques that have been developed in the last decades can be utilized in precision management of hypertension. In particular, non-invasive and simple-to-use techniques, based on PPG, can be implemented in personalized hypertension management.

The personalized management of hypertension aims to increase the precision of the management by adding personal features to the measurement of SBP and DBP. Nevertheless, BP measurement still remains the core of hypertension management, and the precision of the latter is challenged by the low accuracy of the available non-invasive blood pressure measurement techniques. The accuracy of the oscillometric BP measurements can be increased by 24 h ABPM, but routine use of this tool for the general population is impractical, because of its inconvenience and cost. The novel photoplethysmography-based technique for the measurement of systolic blood pressure that was recently found to be more accurate than the two available techniques for BP measurement can be utilized for personalized and more precise management of hypertension.

## Data Availability

Not applicable.
